# Sexual Activity in Peruvian Adolescents: Relevance of Socio-Demographic Variables and Sexual Attitudes

**DOI:** 10.3390/children9030386

**Published:** 2022-03-10

**Authors:** Juan Carlos Sierra, Ana Álvarez-Muelas, Ana Isabel Arcos-Romero, Oscar Cervilla, Pablo Mangas, Laura Elvira Muñoz-García, Fredy S. Monge-Rodríguez

**Affiliations:** 1 Centro de Investigación Mente, Cerebro y Comportamiento (CIMCYC), Universidad de Granada, 18011 Granada, Spain; alvarezm@ugr.es (A.Á.-M.); ocervilla@ugr.es (O.C.); pablomangas@ugr.es (P.M.); noselaura@correo.ugr.es (L.E.M.-G.); 2 Departamento de Psicología, Universidad Loyola, 41704 Sevilla, Spain; aiarcos@uloyola.es; 3 Escuela Profesional de Psicología, Universidad Nacional de San Antonio Abad del Cuzco, Cuzco 08000, Peru; fredy.monge@unsaac.edu.pe

**Keywords:** masturbation, sexual relationships, religiosity, sexual attitudes, Peruvian adolescents

## Abstract

The aim of this study was to explain the masturbation frequency and sexual relationships in adolescents based on age, religious adherence, erotophilia, attitudes toward sexual fantasies and masturbation, and the traditional sexual double standard. A sample of 1120 Peruvian adolescents aged 13–17 years responded to a socio-demographic questionnaire and different scales about sexuality. The frequency of masturbation and sexual relationships, as well as in the sexual attitudes evaluated differed by sex, with boys scoring higher. The masturbation frequency was explained by erotophilia in boys, and by positive attitudes toward sexual fantasies in both sexes. The frequency of sexual relationships was explained by age and positive attitudes toward sexual fantasies in boys and only by the latter in girls. Positive attitudes toward sexual fantasies stand out as a variable for understanding sexual activity in adolescents.

## 1. Introduction

Adolescents are a diverse group with varying needs according to their stage of personal development and life circumstances. As they move from childhood into adolescence all individuals should be provided with the knowledge and skills that will allow them to benefit from the opportunities and overcome the challenges of adulthood [1]. This stage of development is characterized by sexual discovery and experimentation that often begins with precoital behaviours such as kissing, fondling, orogenital contact, and masturbation [2]. Most of the research on adolescent sexual behaviour has focused on the age onset of sexual activity, the prevalence and gender differences of various behaviours, and the rate of sexually transmitted diseases and unwanted pregnancies, but has paid less attention to other behaviours such as masturbation [3]. 

Studying the variables associated with sexual behaviours (e.g., masturbation and sexual relationships) in this life stage is fundamental because, as the World Health Organization indicates, knowledge about the cognitive, emotional, physical, and social aspects of sexuality must be diffused among adolescents through integral sexual education [4]. The adolescence stage is extremely vulnerable in biological, social, and sexual development because immune and reproductive systems are still immature [5]. This stage should be protected by viewing sexual health requirements as the most important public health issue, as there is strong evidence of the positive effects of integral sexual education in increasing adolescents’ knowledge and improving their attitudes toward sexual and reproductive health [6]. According to the World Health Organization [1], these positive effects consist of a delay in the age of sexual onset, a decrease in the frequency of sexual relationships, a reduction in the number of sexual partners and risk-taking, and an increase in the use of condoms and contraceptives. Despite these advances, many adolescents still receive inaccurate, incomplete, or judgement-laden information, leading to the underdevelopment of their physical, social, and emotional capacities [1,4]. Focusing interest on such matters is crucial for the development of healthy and satisfactory sexual behaviours and to prevent risky behaviours [7].

Masturbation is one of the most frequently practiced sexual activities in adolescence [8], and it plays a key role in sexual development that is not substituted by sexual activity with a partner in any life stage [9,10,11]. For example, adult women who masturbated in their adolescence find it easier to become sexually aroused and have orgasms more frequently during sexual relationships than those who did not [12]. Nevertheless, this behaviour is linked to feelings of guilt as a result of negative attitudes toward it [12,13]. Even so, masturbation is present in all life stages [14]. In recent decades, many negative connotations have been disregarded, and masturbation is now considered a beneficial and healthy practice. There is sufficient evidence that it contributes to sexual health [12,15] through body self-learning and sexual response [16]. 

Sexual experiences, especially those early in life, play a crucial role in one’s lifelong sexual course [17]. Moreover, adolescents’ positive beliefs about sexual activity mean that these experiences are part of normative development and, as such, promote suitable adaptation [18]. The frequency of sexual activity has been directly associated with greater sexual satisfaction [19]. 

In 2010 in Peru, the Ministry of Health reported that 19.7% of adolescent secondary education students had experienced sexual relationships and, of these, 46.7% started these practices before reaching the age of 14 years, with higher figures for boys than for girls [20]. Masturbation frequency in Peruvian adolescents is also higher in boys than in girls [14]. According to the 2006 data of the ANAR Foundation’s Free Tele-Advice Service [21], adolescents’ most frequent questions were about sexual relationships and masturbation.

Sexual relationships and masturbation are behaviours influenced by socio-demographic variables (e.g., sex, age, or frequency of religious practice) and by sexual attitudes. Both behaviours appear more frequently in boys than girls [14,22]. In association with age, masturbation frequency increases in boys but remains stable in girls [23]. Another associated variable is the frequency of attending religious events, which delays the start of sexual relationships [24] and has been traditionally associated with sexual guilt caused by masturbation, mostly due to the influence of Jewish-Christian tradition [25].

Sexual attitudes can account for sexual activity by predicting masturbation frequency [26,27] and sexual relationships [28]. Given their link with sexual health, erotophilia, attitudes toward specific sexual behaviours (e.g., masturbation or sexual fantasies), and the traditional sexual double standard stand out among sexual attitudes. Erotophilia is defined as the evaluation of sexual stimuli that can range from a negative valence pole (erotophobia) to its positive valence opposite (erotophilia) [29]. This attitude is strongly associated with the presence of a higher frequency of both masturbation and sexual relationships. Erotophilia was also associated with a higher number of sexual experiences for adolescents [30]. Among specific sexual attitudes, we can highlight positive attitudes toward sexual fantasies. This has been related to interest in both sexual activities with a partner and solitary masturbation [31], which is especially relevant in the adolescence stage when most sexual attitudes are formed [32]. For negative attitudes toward masturbation, a negative association has been observed with masturbation frequency [14,26], which, specifically in adolescents, is an essential factor to help explain feeling sexual guilt, which can affect the frequency with which it is practiced [33]. Finally, the traditional sexual double standard involves more sexual freedom for boys than for girls; thus, boys are expected to engage in more sexual activity [34]. More specifically, in adolescence, peer comparisons take place, so the study of traditional sexual double standards can be relevant [34]. It has also been associated with the age of onset of sexual relationships [34,35,36], with more acceptance of masturbation for boys than for girls [37,38].

Considering this, we aimed to explain the frequency of sexual relationships and solitary masturbation and their correlation with age, frequency of attending religious events, erotophilia, negative attitudes toward masturbation, positive attitudes toward sexual fantasies, and the traditional sexual double standard in Peruvian adolescents. 

According to the evidence, we expected to find differences between boys and girls: (H1) in the frequency of solitary masturbation and sexual relationships, sociodemographic variables (age and frequency of attending religious events), and sexual attitudes (erotophilia, negative attitudes toward masturbation, positive attitudes toward sexual fantasies, and the traditional sexual double standard) [14,39]; and (H2) in the explanatory capacity of these variables on the frequency of both sexual behaviours [33].

## 2. Materials and Methods

### 2.1. Participants

Our study included 1120 Peruvian heterosexual adolescents (559 boys, 561 girls), aged 13 to 17 years (*M* = 14.93; *SD* = 1.39), recruited by non-random sampling. The socio-demographic characteristics of the sample are presented in Table 1. 

### 2.2. Instruments

The socio-demographic questionnaire included questions about sex, age, academic grade, residence, and frequency of attending religious events.

The questionnaire of frequency of both masturbation and sexual relationships consisted of two ad hoc items to assess the frequency of both masturbation and sexual relationships, answered on an 8-point Likert-type scale from 0 (never) to 7 (daily). 

The Spanish version of the Sexual Opinion Survey (SOS-6) [40] evaluates erotophilia with six items answered on a 7-point Likert scale from 1 (totally disagree) to 7 (totally agree). Higher scores indicate higher erotophilia. The internal consistency of the Spanish version was adequate; the ordinal alpha was 0.94 [40]; it presented adequate validity evidence [41]. In this study, the ordinal alpha was 0.58 for boys and 0.53 for girls. 

The Spanish version of the Negative Attitudes Towards Masturbation Inventory (NATMI) [26] consists of 10 items answered on a 5-point Likert-type scale from 1 (totally false) to 5 (totally true). Higher scores indicate a more negative attitude toward masturbation. The ordinal alpha of the Spanish version was 0.95 [26]. In this study, this value was 0.61 for boys and 0.60 for girls. 

The Spanish version of the Hurlbert Index of Sexual Fantasy (HISF) [42] evaluates attitudes toward sexual fantasies. It consists of 10 items answered on a 5-point Likert scale from 0 (never) to 4 (always). Higher scores indicate a more positive attitude toward sexual fantasies. The internal consistency of the Spanish version was 0.94 [42]. In the present study, the ordinal alpha was 0.88 for boys and 0.92 for girls. 

The Spanish version of the Double Standard Scale (DSS) [39] is composed of nine items answered on a 5-point Likert scale from 1 (completely disagree) to 5 (completely agree). Higher scores indicate greater adherence to a traditional sexual double standard. The reliability of the Spanish version was 0.79 [39]. For this study, the ordinal alpha was 0.70 for boys and 0.74 for girls.

### 2.3. Procedure

After conducting a pilot study to validate the content of the questions, two trained interviewers administered the questionnaires in Cuzco from April to July 2011. Participants were recruited from public, secondary education schools by incidental sampling. An all-boys’ school and an all-girls’ school were selected. After providing relevant information to schoolteachers and parents, authorisation and consent forms were given. Questionnaires were administered during class sessions and took 25–30 min to complete. Those students who agreed to participate completed a paper-and-pencil version of the questionnaire in groups of 30–40 within the classroom. They were returned to the researcher in a sealed envelope. Anonymity, confidentiality, and voluntariness from the study were guaranteed. Participants did not receive any form of compensation.

### 2.4. Data Analysis

Firstly, sex differences in all the variables included in the study were examined by calculating Student’s *t*-test and chi-square. The differences for each group were calculated by comparing column proportions. The *p* values for Bonferroni correction were adjusted. Secondly, linear regression-bootstrap models were calculated with the R^®^ environment (version 3.6.3) [43] using its RStudio^®^ interface [44] with the Parameters package (version 0.8.0) [45]. The QuantPsyc package for standardized betas (version 1.4) [46] and the car package for calculating variance inflation factor (VIF) (version 3.0-12) [47] were also employed. The regression analysis was performed separately for boys and girls to explain the frequency of both masturbation and sexual relationships considering the socio-demographic variables and sexual attitudes.

## 3. Results

The results revealed no sex differences in the socio-demographic variables. Regarding sexual attitudes, significant differences were found between boys and girls in erotophilia (24.48 ± 6.30 versus 19.84 ± 6.0; *t* = 12.63, *p* < 0.001), negative attitudes toward masturbation (29.66 ± 5.76 versus 28.48 ± 5.49; *t* = 3.50, *p* < 0.001), positive attitudes toward fantasies (14.31 ± 7.73 versus 5.21 ± 6.22; *t* = 20.10, *p* < 0.001), and the traditional sexual double standard (27.46 ± 5.75 versus 22.52 ± 5.90; *t* = 14.19, *p* < 0.001). Finally, significant differences were observed in the frequencies of both masturbation (*χ*^2^ = 315.86; *p* < 0.001) and sexual relationships (*χ*^2^ = 174.73; *p* < 0.001). In all cases, boys obtained higher scores (Table 2).

After considering the significant differences between boys and girls in the frequency of sexual relationships and masturbation, a decision was made to perform a regression analysis separately by sex. For boys in the masturbation-frequency model, erotophilia (β = 0.20, *p* < 0.001) and positive attitudes toward sexual fantasies (β = 0.30, *p* < 0.001) significantly explained 22% of the variance (*F* = 23.55, *p* < 0.001) in masturbation frequency. For girls, only a positive attitude toward sexual fantasies (β = 0.16, *p* < 0.001) was able to significantly explain 3.3% of the variance (*F* = 3.67, *p* < 0.001) for in masturbation (Table 3).

For boys in the sexual-relationships-frequency model, 16.6% of the variance (*F* = 16.60, *p* < 0.001) was significantly explained by age (β = 0.25, *p* < 0.001) and positive attitudes toward sexual fantasies (β = 0.30, *p* < 0.01). For girls, positive attitudes toward sexual fantasies significantly explained 5.6% of the frequency variance with which sexual relationships took place (*F* = 5.68, *p* < 0.01) (Table 4).

## 4. Discussion

The main objective of this study was to determine the frequency of both sexual relationships and solitary masturbation in Peruvian adolescents considering socio-demographic variables and sexual attitudes. To achieve this, age, frequency of attending religious events, erotophilia, negative attitudes toward masturbation, positive attitudes toward sexual fantasies, and the traditional sexual double standard were considered. 

First, significant differences were found between boys and girls in the frequency of both sexual relationships and masturbation. Boys practiced both behaviours more frequently, which agrees with previous studies reporting similar results [14,22]. Similarly, boys obtained higher scores than girls for the specific evaluated attitudes. Significant differences appeared in attitudes toward sexuality or erotophilia, with more differences in boys than in girls. These findings also coincide with those of previous research works [37,48,49]. Boys showed more positive attitudes toward sexual fantasies, which also agrees with previous studies showing more differences in this attitude for boys [14,42,50,51]. Moreover, boys also showed more negative attitudes toward masturbation, which is congruent with previous studies in Peruvian adolescents [14] and the Spanish population [52,53,54,55]. This may seem contradictory because boys showed more negative attitudes toward masturbation but practised it more frequently. Nonetheless, this finding supports the hypothesis in boys about the compensatory model between masturbation and sexual relationships, which postulates that masturbation may act as a substitute for desired, but not performed, sexual relationships [11,54,55,56]. A negative attitude toward masturbation showed explanatory capacity in feelings of sexual guilt in adolescents [33]. From this perspective of masturbation as a compensatory behaviour, higher practice may be associated with more negative feelings. For example, compared to girls, boys showed more personal negative affect in relation to masturbation [14]. Attitudes toward the traditional sexual double standard were more present in boys than girls, which coincides with other studies describing the same differences in a Spanish [57,58] and a Salvadoran population [59].

Then, explanatory models were examined for the frequency of both masturbation and sexual relationships. Masturbation frequency in boys was explained positively by erotophilia, along with showing a positive attitude toward sexual fantasies. In girls, however, only positive attitudes toward sexual fantasies positively explained masturbation frequency. Erotophilia is a relevant variable in the sexual experiences of adolescents [30]. In this study, boys showed more erotophilia than girls according to previous research [40,48,49], so it is not surprising that this variable was more relevant in explaining boys’ masturbation frequency. Regarding attitudes toward sexual fantasies for boys and girls, these results indicated their role in relation to masturbation [14,26,60], where the positive attitude were associated with higher masturbation frequency [61]. 

Additionally, sexual relationships frequency was explained positively by age and positive attitudes toward sexual fantasies in boys, but only by positive attitudes toward sexual fantasies in girls. In boys, as age increases, the frequency of sexual relationships appears to increase. Its role could indicate sex differences in psycho-evolutionary development and social norms in adolescence. A positive attitude toward sexual fantasies, which played a positive role in boys and girls, confirmed previous findings, which indicated a relation between a positive attitude toward sexual fantasies and an interest in sexual activity [42]. This stresses the role of specific attitudes, particularly positive attitudes toward sexual fantasies, as a relevant variable in sexual health [54].

This study has its limitations. Despite its large sample size, the participants were recruited by nonprobabilistic incidental sampling and the inclusion criterion was sexual orientation. These matters must be considered to interpret and generalise the results, and to continue this study with a more diverse sample. 

## 5. Conclusions

This work provides relevant findings about the frequency of both solitary masturbation and sexual relationships in adolescents in research, education, and clinical areas. Differences exist between the sexual attitudes of boys and girls. Boys show higher erotophilia, negative attitudes toward masturbation, positive attitudes toward sexual fantasies, and the traditional sexual double standard. In relation to the explanatory capacity of masturbation and sexual behaviours, the role of positive attitudes toward sexual fantasies stands out, which confirms its importance for sexual health and it being considered in programmes that promote and intervene in the adolescent population’s sexual functioning.

## Figures and Tables

**Table 1:** Sociodemographic characteristics of the sample.

	Total *M* (*SD*)/*n* (%)	Boys *M* (*SD*)/*n* (%)	Girls *M* (*SD*)/*n* (%)
Age	14.93 (1.39)	14.91 (1.4)	14.94 (1.4)
Academic grade			
1st	14 (1.3%)	4 (0.7%)	10 (1.8%)
2nd	266 (23.8%)	128 (22.9%)	138 (24.6%)
3rd	223 (19.9%)	152 (27.2%) ^a^	71 (12.7%) ^b^
4th	285 (25.4%)	105 (18.8%) ^a^	180 (32.1%) ^b^
5th	330 (29.6%)	170 (30.4%)	162 (28.9%)
Residence			
Urban	967 (86.3%)	481 (86%)	486 (86.6%)
Rural	153 (13.7%)	78 (14%)	75 (13.4%)

Note: Different superscript letters (^a^, ^b^) denote the proportions of groups that significantly differed. *M* = mean; *SD* = standard deviation.

**Table 2:** Differences between the sexes in the observed variables.

	Boys (*n* = 559) *M* (*SD*)/*n* (%)	Girls (*n* = 561) *M* (*SD*)/*n* (%)	*t*/*χ*^2^	Cohen’s d
Age	14.91 (1.4)	14.94 (1.4)	−0.43	
Religious frequency				
Never	54 (9.7%)	33 (5.9%)	6.09	
One a year	93 (16.6)	104 (18.5%)		
Once a month	106 (19%)	106 (18.9%)		
Once a week	299 (53.5%)	309 (55.1%)		
Daily	7 (1.3%)	9 (1.6%)		
Erotophilia	24.48 (6.30)	19.84 (6)	12.63 ***	0.75
Negative attitudes toward masturbation	29.66 (5.76)	28.48 (5.49)	3.50 ***	0.21
Positive attitudes toward sexual fantasies	14.31 (7.73)	5.21 (6.22)	20.10 ***	1.30
Traditional sexual double standard	27.46 (5.75)	22.52 (5.90)	14.19 ***	0.85
Masturbation frequency				
Never	228 (40.8%) ^a^	507 (90.4%) ^b^	315.86 ***	
A few times a year	132 (23.6%) ^a^	35 (6.2%) ^b^		
Once a month	77 (13.8%) ^a^	16 (2.9%) ^b^		
Two or three times a month	45 (8.1%) ^a^	2 (0.4%) ^b^		
Once a week	29 (5.2%) ^a^	0 (0%) ^b^		
Two or three times a week	29 (5.2%) ^a^	0 (0%) ^b^		
Four to six times a week	5 (0.9%) ^a^	0 (0%) ^b^		
Daily	14 (2.5%) ^a^	1 (0.2%) ^b^		
Sexual relationships frequency			174.73 ***	
Never	367 (66.5%) ^a^	541 (97%) ^b^		
A few times a year	115 (20.8%) ^a^	14 (2.5%) ^b^		
Once a month	24 (4.3%) ^a^	3 (0.5%) ^b^		
Two or three times a month	18 (3.3%) ^a^	0 (0%) ^b^		
Once a week	15 (2.7%) ^a^	0 (0%) ^b^		
Two or three times a week	5 (0.9%) ^a^	0 (0%) ^b^		
Four to six times a week	2 (0.4%)	0 (0%)		
Daily	6 (1.1%) ^a^	0 (0%) ^b^		

Note: Different superscript letters (^a^, ^b^) denote the proportions of groups that significantly differed. *** *p* < 0.001. For sexual relationships frequency, the sample was composed of 552 boys and 558 girls.

**Table 3:** Multiple regression models for masturbation frequency in boys and girls.

Predictors	B	SE	β	95% CI	*t*	*p*	*R* ^2^	VIF
Boys (*n* = 559)							0.22	
Sexual relationships frequency	−0.02	0.06	−0.02	[−0.14, 0.13]	−0.39	0.699		1.21
Age	−0.09	0.05	−0.07	[−0.21, 0.02]	−1.82	0.069		1.17
Religious frequency	−0.04	0.06	−0.02	[−0.16, 0.07]	−0.61	0.541		1.08
Erotophilia	0.05	0.01	0.20	[0.03, 0.08]	4.44	<0.001		1.43
Negative attitudes toward masturbation	−0.02	0.01	−0.07	[−0.04, 0.00]	−1.79	0.073		1.09
Positive attitudes toward sexual fantasies	0.06	0.01	0.30	[0.04, 0.08]	6.21	<0.001		1.64
Traditional sexual double standard	0.02	0.01	0.07	[−0.01, 0.05]	1.61	0.108		1.30
Girls (*n* = 561)							0.03	
Sexual relationships frequency	0.15	0.11	0.06	[−0.05, 0.42]	1.45	0.149		1.07
Age	−0.02	0.02	−0.06	[−0.05, 0.00]	−1.52	0.129		1.06
Religious frequency	−0.02	0.02	−0.05	[−0.08, 0.02]	−1.15	0.252		1.02
Erotophilia	−0.00	0.00	−0.03	[−0.01, 0.00]	−0.68	0.494		1.22
Negative attitudes toward masturbation	0.00	0.00	0.02	[−0.00, 0.01]	0.58	0.559		1.06
Positive attitudes towards sexual fantasies	0.01	0.00	0.16	[0.01, 0.02]	3.50	<0.001		1.29
Traditional sexual double standard	0.01	0.00	0.07	[0.00, 0.02]	1.73	0.085		1.07

Note: VIF = Variance inflation factor.

**Table 4:** Multiple regression models for sexual relationships frequency in boys and girls.

Predictors	B	SE	β	95% CI	*t*	*p*	*R^2^*	VIF
Boys (*n* = 552)							0.17	
Masturbation frequency	−0.01	0.03	−0.02	[−0.09, 0.06]	−0.39	0.699		1.30
Age	0.22	0.04	0.25	[0.15, 0.30]	6.06	<0.001		1.10
Religious frequency	−0.01	0.05	−0.01	[−0.12, 0.09]	−0.19	0.849		1.08
Erotophilia	−0.00	0.01	−0.00	[−0.02, 0.02]	−0.10	0.916		1.48
Negative attitudes towards masturbation	−0.01	0.01	−0.05	[−0.03, 0.01]	−1.30	0.194		1.09
Positive attitudes toward sexual fantasies	0.04	0.01	0.30	[0.03, 0.06]	6.00	<0.001		1.65
Traditional sexual double standard	0.01	0.01	0.06	[0.00, 0.04]	1.41	0.160		1.30
Girls (*n* = 558)							0.06	
Masturbation frequency	0.02	0.02	0.06	[0.00, 0.09]	1.45	0.149		1.04
Age	0.01	0.01	0.07	[0.00, 0.02]	1.73	0.084		1.06
Religious frequency	−0.02	0.01	−0.07	[−0.04, 0.00]	−1.80	0.073		1.01
Erotophilia	−0.00	0.00	−0.02	[0.00, 0.00]	−0.49	0.624		1.22
Negative attitudes towards masturbation	−0.00	0.00	−0.01	[0.00, 0.00]	−0.16	0.871		1.06
Positive attitudes toward sexual fantasies	0.01	0.00	0.20	[0.00, 0.01]	4.29	<0.001		1.28
Traditional sexual double standard	0.00	0.00	0.03	[0.00, 0.01]	0.70	0.486		1.07

Note: VIF = Variance inflation factor.

## Data Availability

The data presented in this study are available on request from the corresponding author. The data are not publicly available due to privacy.
